# Editorial: Pathogens and Pregnancy Failure in Domestic Animals

**DOI:** 10.3389/fvets.2022.812150

**Published:** 2022-02-10

**Authors:** Maria Rosa Caro

**Affiliations:** Animal Health Department, University of Murcia, Murcia, Spain

**Keywords:** Brucella, Chlamydia, pestivirus, Neospora, Toxoplasma, PCRs, pregnancy, abortion

Infection is a major cause of pregnancy failure in eutherian mammals. The reproductive pathogens are diverse and can be either viral, bacterial, fungal, or protozoan. There is a direct negative impact on pregnancy and, in the case of farm livestock, sustainability is compromised, leading to significant economic loss. These novel contributions to the latest research in the field of reproductive pathology represent a major breakthrough for the improvement of animal and human health within the “One Health” concept.

The contributions were six articles published within the topic totaling more than 103,000 views to date.

Montbrau et al. from Spain reported the efficacy of a new commercially available inactivated vaccine against ovine enzootic abortion (OEA) produced by *Chlamydia abortus*, an intracellular bacterium that causes strong economic losses in the small ruminants industry worldwide. The authors suggest that this vaccine is adequate for the control and prevention of OEA, suggesting further future studies to elucidate the type of cellular protective immune response induced by the vaccine.

Santos et al. from Brazil showed a brief, but very current review, on canine brucellosis, an infectious and zoonotic disease caused by *Brucella canis*. The authors emphasized the importance of completing the serological diagnosis with molecular methods as well as the development of safe vaccines necessary to control canine brucellosis and its associated risk of zoonosis.

Decaro from Italy presented an excellent update on HoBi-like Pestivirus, an emerging group of pestiviruses that are detected in cattle and other ruminants in South America, Europe, and Asia, where they cause serious reproductive disorders in these species.

Wolf-Jäckel et al. from Denmark, using molecular methods such as polymerase chain reaction (PCR), next-generation sequencing, real-time quantitative 16S rDNA PCR (qPCR) amplicon sequencing, and FISH, found that these molecular methodologies are good alternatives to traditional microbial culturing methods. The authors identified several bacterial and fungal abortifacients from aborted or stillborn bovine fetuses and placentas.

In Spain, Pastor-Fernández et al. reviewed the interactions between placental host-apicomplexan parasites such as *Neospora caninum* and *Toxoplasma gondii*, the etiological agents of bovine neosporosis, and sheep and goat toxoplasmosis, respectively. The authors described the interactions and the importance of an alternative to improve the *in vitro* systems based on caruncular and trophoblast cells. The use of placental explants is widely developed in human research. The authors discussed *in vivo, in vitro*, and *ex vivo* placental models that have proven useful to unravel the pathogenic mechanisms and the host immune response responsible for fetal death caused by neosporosis and toxoplasmosis.

The editors are convinced that you will find in this Research Topic stimulating, and that the information may generate new ideas for research.

As a personal experience, it has been a pleasure to work as a co-editor with the journal Frontiers in Veterinary Sciences and I encourage my colleagues to participate in similar actions that can contribute to the transfer of knowledge on reproductive science in the veterinary medicine area.

## Author Contributions

The author confirms being the sole contributor of this work and has approved it for publication.

## Conflict of Interest

The author declares that the research was conducted in the absence of any commercial or financial relationships that could be construed as a potential conflict of interest.

## Publisher's Note

All claims expressed in this article are solely those of the authors and do not necessarily represent those of their affiliated organizations, or those of the publisher, the editors and the reviewers. Any product that may be evaluated in this article, or claim that may be made by its manufacturer, is not guaranteed or endorsed by the publisher.

